# Elevated Neutrophil Lymphocyte Ratio in Recurrent Optic Neuritis

**DOI:** 10.1155/2015/758687

**Published:** 2015-04-28

**Authors:** Hande Guclu, Sadık Altan Ozal, Vuslat Pelitli Gurlu, Ramazan Birgul

**Affiliations:** Department of Opthalmology, Faculty of Medicine, Trakya University, 22030 Edirne, Turkey

## Abstract

*Purpose*. To demonstrate the relation between optic neuritis (ON) and systemic inflammation markers as neutrophil lymphocyte ratio (N/L ratio), platelet count, mean platelet volume (MPV), and red cell distribution width (RDW) and furthermore to evaluate the utilization of these markers to predict the frequency of the ON episodes. *Methods*. Forty-two patients with acute ON and forty healthy subjects were enrolled into the study. The medical records were reviewed for age, sex, hemoglobin (Hb), Haematocrit (Htc), RDW, platelet count, MPV, white blood cell count (WBC), neutrophil and lymphocyte count, and neutrophil lymphocyte ratio (N/L ratio). *Results*. The mean N/L ratio, platelet counts, and RDW were significantly higher in ON group (*p* = 0.000, *p* = 0.048, and *p* = 0.002). There was a significant relation between N/L ratio and number of episodes (*r* = 0.492, *p* = 0.001). There was a statistically significant difference for MPV between one episode group and recurrent ON group (*p* = 0.035). *Conclusions*. Simple and inexpensive laboratory methods could help us show systemic inflammation and monitor ON patients. Higher N/L ratio can be a useful marker for predicting recurrent attacks.

## 1. Introduction

Optic neuritis (ON) is an acute demyelinating inflammatory disease of the optic nerve and primarily influences young females [1, 2]. It is usually related to multiple sclerosis (MS) or neuromyelitis optica (NMO) or can be seen isolated [2]. It is observed after paranasal sinuses infections or viral infections [3–5]. First attack of ON occurs 70% unilaterally and 30% bilaterally of the patients in the third decade of life [6]. ON's incidence is 1–5 in 100,000 population in every year [6, 7]. Clinical criteria to diagnose ON are acute visual loss, dyschromatopsia, and pain of the eye [8]. In addition, reduced detection of light brightness and loss of vision can be provoked by increased body temperature or exercise and stereo illusion when looking at a swinging pendulum after a neutralization filter [9].

Systemic inflammation is detectable with many biochemical and hematological tests. However, specific tests are quite expensive and complicated. Neutrophil lymphocyte ratio (N/L ratio) is an effective, easy, and inexpensive method to determine systemic inflammation in various diseases such as cancer, hypertension, diabetes mellitus, and coronary artery disorders [10, 11]. Platelet activation occurs in inflammatory diseases and mean platelet volume (MPV) is a marker for it. Increased platelet count revealed the bone marrow activity [12–14]. Red cell distribution width (RDW) is size variation of the red blood cells and also an inflammatory marker for some diseases [15–19].

We aimed to demonstrate the relation between ON and systemic inflammation markers as N/L ratio, platelet count, MPV, and RDW and furthermore to evaluate the utilization of these markers to predict the frequency of the ON episodes.

## 2. Materials and Methods

Records of the 106 patients admitted with ON in our clinic between the years 2006 and 2014 were reviewed retrospectively. Forty-two patients with acute ON whose blood samples were received before treatment with intravenous corticosteroids and randomly selected forty healthy subjects (age and sex matched) were enrolled into the study. Each patient was monitored at least for 12 months. All subjects underwent full ophthalmological examination including best-corrected visual acuity (BCVA), relative afferent pupillary defect, slit lamp, and fundus examination. In addition, a complete neurological examination, cranial magnetic resonance imagining, visuel field analyze and visual evoked potential were also performed. All patients were treated with intravenous corticosteroids (methylprednisolone 4 × 250 mg/kg per day) for three days and continued with oral prednisone 1 mg/kg for 15 days after that corticosteroid medication reduced and stopped. Two or more ON attacks in the same eye or other eye appearing in different period were assigned as recurrent ON.

The medical records were reviewed for age, sex, hemoglobin (Hb), Haematocrit (Htc), RDW, platelet count, MPV, white blood cell count (WBC), neutrophil and lymphocyte count, N/L ratio, which is identified as dividing neutrophil count to lymphocyte count; blood parameters were measured by fluorescent flow cytometry method using the Sysmex XE-2100 (Sysmex Corporation, Kobe, Japan) automated hematology system. Subjects' blood samples were received in the dipotassium ethylenediaminetetraacetic acid (EDTA) tubes.

Patients who have smoking habits, infection, chronic diseases such as diabetes mellitus, hypertension, cancer, liver disorders, kidney disorders, hematological diseases, coronary artery diseases, inflammatory diseases such as ankylosing spondylitis, Behçet's disease and rheumatoid arthritis, history of any medications, and ocular diseases were excluded from the study.

The procedures of the study were approved by the institutional review board of the hospital and adhered to the tenets of the Declaration of Helsinki.

### 2.1. Statistical Analysis

Statistical analysis was performed using SPSS (version 22, SPSS Inc, IL). All results are presented as mean standard error of the mean. The independent Student's *t*-test was used to compare data between patient subgroups. Student's *t*-test, used to determine normally distributed data, and the Mann-Whitney *U* test, used to detect data that do not comply with an average distribution, were used to compare data between two groups. To compare nonparametric data Chi-square test was used. The Spearman test was used to assess the correlation between variables. ROC (Receiver Operating Characteristics) curve of N/L ratio was used to discriminate recurrence in optic neuritis patients. *p* < 0.05 was assumed significant for all analysis.

## 3. Results

This study includes 42 ON patients and 40 healthy subjects. Mean age of the ON patients is 30.81 ± 10.7 years (13–52), and healthy subjects are 28.9 ± 10.1 years (13–50). The mean age and sex were not significantly different between patients and healthy groups (*p* = 0.423, *p* = 0.320) (Table 1). Mean follow-up period was 25.8 ± 21 (12–108) months. ON patients had significantly higher white blood cell and neutrophil count (*p* = 0.001, *p* = 0.000). For lymphocyte counts, we did not find any statistically significant difference between patients and healthy group (*p* = 0.241). The mean N/L ratio was significantly higher in ON group (*p* = 0.000). There was a significant difference between platelet counts of patients and control group (*p* = 0.048). The mean MPV was lower in ON group, but the difference was not found to be significant (*p* = 0.241). The mean Hb and Htc were significantly lower in patients group, but laboratory results were within normal limits (*p* = 0.000, *p* = 0.000). RDW was significantly higher in ON group than the control group (*p* = 0.002).

Twenty patients had at least two or more ON attacks. The mean age of ON patients with one episode was 29.4 ± 10.6, and the mean age of recurrent ON patients was 32.3 ± 10.9. Patients with recurrent ON have higher N/L ratio. There was a significant relation between N/L ratio and number of episodes (*r* = 0.492, *p* = 0.001). There was a statistically significant difference for MPV between one episode group and recurrent ON group (*p* = 0.035) (Table 2).

Number of episodes in the patients with white matter demyelination in cranial MRI (2.0 ± 0.7) was significantly higher than the patients with normal cranial MRI (1.3 ± 0.6) (*p* = 0.006). Significant positive correlation was found between white matter demyelination in cranial MRI and number of episodes (*r* = 0.430, *p* = 0.005), according to this correlation when the white matter demyelination in cranial MRI increases the number of episodes increases too. No significant difference was detected between age of recurrent ON and one episode group (*p* = 0.399). Sixteen of the patients had been diagnosed with MS. WBC and neutrophil count were found significantly increased in MS patients (*p* = 0.009, *p* = 0.032). N/L ratio was 2.75 ± 1.63 in clinically definite MS patients and 2.68 ± 1.52 in the patients with no clinically definite MS. No significant difference was determined for N/L ratio (*p* = 0.885), lymphocyte count (*p* = 0.076), platelet count (*p* = 0.360), RDW (*p* = 0.929), and MPV (*p* = 0.310) between patients who had not been diagnosed with MS and MS patients. Fifteen of the patients were female, 7 were male in one episode group, and there was no significant difference between two groups (*p* = 0.088). Sixteen of the patients were female, and 4 were male in recurrent ON group but there was a significant difference between two groups (*p* = 0.007).

**Figure 1:**
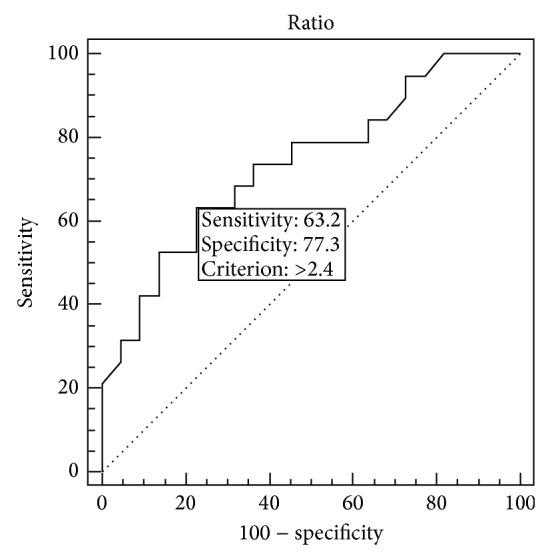
ROC curve of N/L ratio to discriminate recurrence in optic neuritis patient.

Twenty-two patients had just one ON attack. When we compare the patients with one ON episode and healthy subjects; a significant difference was found in neutrophil count (*p* = 0.001) but no significant difference was found for lymphocyte count and N/L ratio (*p* = 0.697, *p* = 0.059). Platelet count and RDW were significantly higher, and MPV was lower in the patients with one ON attack than the control group (*p* = 0.009, *p* = 0.012, and *p* = 0.047). There was a statistically significant difference in N/L ratio between the control group and recurrent ON group. N/L ratio was found statistically higher in recurrent ON group than the control group (*p* = 0.000). ROC curve analysis of N/L ratio was performed to discriminate the recurrence in ON patients (Figure 1). Area under the curve (AUC) of N/L ratio to discriminate recurrence in ON patients was found as 0.738 (*p* = 0.002), and a cut-off point was determined >2.4. Then, sensitivity was calculated as 63.2%, and specificity was calculated as 77.3% at the cut-off point.

## 4. Discussion

Optic neuritis is one of the most common causes of acute visual loss in the young population [2, 20]. Visual acuity spontaneously increases over days and weeks, but the improvement is completed almost in 2 or 3 months [21]. Even if the visual acuity is 20/20, some patients have decreased colour vision and contrast sensitivity, reduced stereo acuity, visual field deficits, relative afferent pupillary defect, optic disc abnormalities, and impaired visual evoked potentials [21–24].

Optic neuritis is the initial manifestation in 20% of MS patients [20, 25]. While attack rate is 30% in isolated ON patients, MS patients have 50% attack rate within five years [26]. It was found that intravenous corticosteroids supply faster visual recovery, but they are not effective to the degree of permanent visual loss. Also, usage of oral corticosteroids alone is inefficient to improve visual acuity and is related with increased attack frequency [21, 25, 27]. Pathophysiology of recurrent ON is still not clear [28]. Patients with demyelinating lesions tend to develop MS when compared with the patients without demyelinating lesions [29]. The patients with NMO have frequent attack rate too. NMO-immunoglobulin G stated higher in recurrent ON patients [28]. Aquaporin 4 antibody positive patients are more disposed to have higher recurrence of serious ON [30]. However, there is no unique marker predicting the frequency of the ON episodes. Most of the studies reported expensive and quite complex methods to demonstrate it. Recently several studies recommended high N/L ratio as an indicator for inflammatory diseases, psoriasis, acute coronary syndrome, diabetes, and cancer [10, 11, 31]. Imtiaz et al. reported that N/L ratio was utilized as a prognostic marker in cancer and coronary artery bypass patients [10]. Ilhan et al. studied N/L ratio for age-related macular degeneration and reported high N/L ratio. Karaca et al. found increased N/L ratio in progressive keratoconus patients [32, 33]. Multiple studies researched N/L ratio in diabetic retinopathy [34–36]. In present study N/L ratio was higher in ON patients than the control group. The N/L ratio was also significantly higher in patients who had recurrent attacks during the follow-up time. N/L ratio can be a useful, inexpensive, and easy marker to predict that a patient could have recurrent ON attack, and we consider that N/L ratio could show the disease activity. In present study, there is a relation between white matter demyelinating abnormalities and recurrent ON. Patients with white matter abnormalities on cranial MRI have increased number of episodes and beside this when the number of demyelinating lesions increases on cranial MRI attack rate increases too. Du et al. demonstrated that patients with white matter demyelinating lesions tend to develop MS and MS patients have more frequent attack rate [28]. We found higher N/L ratio in ON patients with clinically definite MS than the patients with no clinically definite MS but also there was no statistically significant difference between two groups.

Platelet count and MPV are related to platelet activation [12]. MPV has an inverse correlation with acute, active inflammation and referred as a negative acute phase reactant [37, 38]. In our study MPV was lower in patients with ON than the control group; it was not significant, but increased platelet count was statistically significant. Recurrent ON group had significantly elevated MPV and lower thrombocyte count than patients with just one episode; acute ON exacerbation upon ongoing chronic inflammation could be the reason for these laboratory results. The first episode stimulates acute, significantly increased inflammatory status that could be related to decreased MPV and elevated platelet count in patients with one episode group.

Inflammation interrupts production and activity of erythropoietin, thus destroying the erythrocyte maturation, resulting in immature erythrocytes and increased RDW [39]. Previous studies determined that RDW is positively correlated with inflammation, and it shows disease activity in systemic lupus erythematosus, rheumatoid arthritis, and inflammatory bowel diseases [19, 40–42]. In present study, ON patients have higher RDW than healthy subjects but there was no difference for RDW between one episode and recurrent group.

The limitation of our study is its retrospective design; even if the present study has relatively small sample size, it is the first study that demonstrates the relation between systemic inflammation and ON. Design of the study creates bias problem as a general difficulty in all retrospective studies. Potential bias was removed by random assignment of control groups, blinding data collectors to the study hypothesis. Also, all ON patients whose blood samples were received before the treatment enrolled into the study. We believe that our study can encourage researchers to investigate systemic inflammation markers in ON patients by prospective multicentric randomised clinical studies.

In conclusion, simple and inexpensive laboratory methods could help us show systemic inflammation and monitor ON. Higher N/L ratio can be a guide for predicting recurrent attacks, but further prospective studies with the high number of patients and long follow-up time are needed.

## Figures and Tables

**Table 1:** Demographic characteristics and laboratory results of patients and control groups.

	Optic neuritis	Control	*p*
Age (years)	30.81 ± 10.7	28.9 ± 10.1	0.423
Sex (female/male)	22/20	20/20	0.320
WBC (/mm^3^)	8381 ± 1885	7048 ± 1471	0.001
NEU (/mm^3^)	5387 ± 1684	3.893 ± 1010	0.000
LYMP (/mm^3^)	2218 ± 723	2404 ± 700	0.241
N/L ratio	2.71 ± 1.5	1.70 ± 0.5	0.000
PLT (/mm^3^)	274000 ± 55550	249000 ± 60332	0.048
MPV (f/L)	7.94 ± 0.97	8.21 ± 1.15	0.241
RDW (%)	14.1 ± 1.27	13.3 ± 1.00	0.002

WBC: white blood cell, NEU: neutrophil, LYMP: lymphocyte, PLT: platelet, RDW: red cell distribution width, and N/L: neutrophil lymphocyte ratio.

**Table 2:** Demographic characteristics and laboratory results of one episode optic neuritis patients and recurrent optic neuritis groups.

	Patients with one optic neuritis episode	Recurrent optic neuritis	*p*
Age (years)	29.45 ± 10.6	32.3 ± 10.19	0.399
WBC (/mm^3^)	8199 ± 1639	8581 ± 2149	0.519
NEU (/mm^3^)	4955 ± 1312	5863 ± 1940	0.081
LYMP (/mm^3^)	2478 ± 738	1933 ± 601	0.013
N/L ratio	2.06 ± 0.8	3.42 ± 1.8	0.003
PLT (/mm^3^)	288630 ± 53191	259750 ± 55421	0.093
MPV (f/L)	7.64 ± 0.93	8.27 ± 0.93	0.035
RDW (%)	14.2 ± 1.30	14.0 ± 1.26	0.606

WBC: white blood cell, NEU: neutrophil, LYMP: lymphocyte, PLT: platelet, RDW: red cell distribution width, and N/L ratio: neutrophil lymphocyte ratio.
